# Emerging presence of *Actinomyces* in perianal and pilonidal infection

**DOI:** 10.1093/jscr/rjac367

**Published:** 2022-08-17

**Authors:** Chih Y Tan, Muhammad A Javed, Dmitri Y Artioukh

**Affiliations:** Department of General Surgery, Cambridge University Hospital, Cambridge, UK; Liverpool University Hospitals NHS Foundation Trust, Liverpool, UK; Department of General Surgery, Southport and Ormskirk Hospital NHS Trust, Southport, UK

## Abstract

*Actinomyces* is a rare aetiology of infections affecting the perianal region and natal cleft. Recognition of this microorganism is essential to deliver targeted antimicrobial therapy following surgical intervention. We present a series of 15 pilonidal and perianal infections associated with this microorganism. *Actinomyces turicensis* was the only strain of Actinomyces isolated. A total of 14 out of 15 cases had concomitant microorganisms isolated from microbiology specimens. Mixed anaerobes (*n* = 14) were the most common concomitant pathogens followed by *Streptococcus milleri* (*n* = 3), *Staphylococcus aureus* (*n* = 1), *Citrobacter* (*n* = 1) and *Coliform* bacteria (*n* = 1). All patients, except one who was pregnant at time of diagnosis, underwent surgical drainage with or without further oral antibiotic therapy. Coloproctologists need to consider *Actinomyces* as a clinically significant pathogen in the context of perianal and pilonidal infections.

## INTRODUCTION

Actinomycosis is a rare, chronic infection caused by anaerobic gram-positive bacteria, Actinomyces spp. Over 30 species have been isolated, largely as commensal flora in the oral cavity, urogenital tract and gastrointestinal tract [1]. *Actinomyces turicensis* was first identified by Wüst *et al*. in 1995 by using 16S ribosomal RNA gene sequencing technique [2] and has since been described as potential pathogen mostly involving genital, skin-related and urinary tract infections [3]. To date, there have been only a handful of reports of perianal and natal cleft infections caused by *A. turicensis*. Recognition of this pathogen is of paramount importance for successful eradication of infection by target antimicrobial therapy after appropriate surgical intervention. We present a series of perianal and pilonidal infections associated with this microorganism.

## CASE SERIES

Retrospective review of clinical and microbiological databases identified 15 patients who presented with perianal and natal cleft sepsis and had Actinomyces spp. isolated from microbiological specimens between 2013 and 2015. There were eight females and seven males with the median age of 22 years (range: 19–52) at the time of the diagnosis. *Actinomyces turicensis* was the only strain of *Actinomyces* identified in all patients in addition to a number of additional microorganisms. Mixed anaerobes were most common (*n* = 14), followed by *Streptococcus milleri* (*n* = 3), *Staphylococcus aureus* (*n* = 1), *Citrobacter* (*n* = 1) and *Coliform* bacteria (*n* = 1). In one patient, *A. turicensis* was the only pathogen isolated from the wound. All patients except one who was pregnant were managed by surgical drainage of abscesses with or without antibiotic therapy of variable duration (Table 1). This observational study did not allow to conclude whether *A. turicensis* was the primary microorganism responsible for perianal and pilonidal sepsis or simply concomitant pathogen of no clinical significance. Its isolation, however, in cases of non-healing discharging surgical wounds necessitated a long course of antibiotics up to 3 months duration to achieve resolution of symptoms.

**Table 1:** Clinical summary of cases that isolated *Actinomyces spp.*

Patient	Age	Gender	Concomitant microorganisms	Location of infection	Antibiotics and duration of treatment
1	25	Female	Nil	Pilonidal	Augmentin (12 days)
2	20	Male	Mixed anaerobes *Streptococcus milleri*	Pilonidal	4× courses (regime unknown)
3	24	Male	Mixed anaerobes	Pilonidal	Nil
4	21	Female	Mixed anaerobes	Pilonidal	Augmentin (5 days)
5	27	Female	Mixed anaerobes	Pilonidal	Nil
6	19	Male	Mixed anaerobes	Pilonidal	Nil
7	21	Female	Mixed anaerobes	Pilonidal	Augmentin (7 days)
8	19	Female	Mixed anaerobes *Streptococcus milleri*	Pilonidal	Nil
9	52	Male	Mixed anaerobes	Pilonidal	Augmentin (5 days)
10	15	Female	Mixed anaerobes *Citrobacter* *Staphylococcus aureus*	Pilonidal	Amoxicillin (3 months)
11	23	Male	Mixed anaerobes	Pilonidal	Nil
12	28	Female	Mixed anaerobes	Perianal	Nil
13	20	Female	Mixed anaerobes *Coliform* *Streptococcus milleri*	Perianal	Amoxicillin + metronidazole (2 weeks), amoxicillin (3 months)
14	24	Male	Mixed anaerobes	Perianal	Nil
15	22	Male	Mixed anaerobes	Perineal	Nil

## DISCUSSION

Actinomycosis is a rare, indolent and chronic bacterial infection that exerts both suppurative and granulomatous inflammation. It is caused by filamentous gram-positive anaerobic bacteria from the *Actinomycetaceae* family [4]. More than 30 species had been identified and *Actinomyces israelii* being the most common human pathogen. The condition is classified into distinct clinical forms based on the anatomical location of the inflammatory process. Orocervical infection being the most common form, which accounted for 50% reported cases, followed by thoracic actinomycosis (15–20%) and abdominopelvic actinomycosis (20%). Rare sites of infection include central nervous system, musculoskeletal tissues and prosthetic joints [4].

Diagnosis of actinomycosis requires demonstration of sulphur granules on histological examination and direct isolation of filamentous gram-positive anaerobic bacteria. However, presence of sulphur granules is highly suggestive of actinomycosis but non-specific as they are also observed in other infections, notably nocardiosis [4]. Isolation of actinomycosis can be challenging and failure rate is high (>50%) due to several reasons, including previous antibiotics treatment, culture in anaerobic conditions, slow growth (up to 3 weeks), selective agar medium at optimal 37°C and overgrowth of concomitant bacteria [4, 5].

As with other forms of infection, antibiotics are required to treat actinomycosis. Historically, high-dose intravenous Penicillin G is the medication of choice during initial 1–2 weeks followed by oral penicillin for a prolonged period from 6 to 12 months [6]. Although antibiotic therapy is the mainstay treatment for actinomycosis, surgical intervention may be indicated for drainage of abscess, obtaining biopsy and treatment of sinus or fistula. Thus, six patients in our series received penicillin-based antibiotic therapy.

Similar to findings of Sabbe *et al*. and Wüst *et al*., we observed association between *A. turicensis* and *S. milleri* that co-existed in 3 out 15 of our specimens. The reason for this affinity remains uncertain, but comparable properties of both pathogens and the same habitat have been discussed among the causes [3, 7].

In conclusion, *Actinomyces spp.* may be an emerging pathogen in perianal and natal cleft infections and should not be seen as clinically insignificant co-infectors or simply contaminants. Clinical awareness of the possibility of this infection, appropriate microbiological testing and antibiotic therapy will improve outcomes, particularly in patients with persistent and recurrent sepsis.

## AUTHORS’ CONTRIBUTIONS

All authors contributed to the study conception and design. Material preparation, data collection and analysis were performed by C.Y.T. and D.Y.A. The first draft of the manuscript was written by C.Y.T. and all authors commented on previous versions of the manuscript. All authors read and approved the final manuscript.

This article was presented in poster format at the Association of Surgeon in Training (ASIT) conference 2016 and Association of Surgeons of Great Britain and Ireland (ASGBI) conference 2016.
